# On Spinner's Phthisis

**Published:** 1831

**Authors:** 


### XVII.

### On Spinner's Phthisis. By Dr. Kay, of Manchester.

Dr. Kay has written a long and important article "on molecular irritation of the lungs, as one cause of tubercular consumption," in the third Number of the North of England Journal, from which we shall make a few extracts. A considerable portion of it is occupied with observations and criticisms on the writings of Velpeau, Broussais, Alison, Andral, Baron, and others; together with personal experiments on the introduction of mercury into the lungs—experiments quoted in Dr. Alison's work, they having been made while Dr. Kay was an "*interne*" in the Royal Infirmary of Edinburgh. But these critiques and experiments we must pass over, in order to come at once to the principal and practical part of the communication.

The situation of the Ardwick Dispensary, in the centre of a dense mass of cotton-spinners, has afforded our author ample opportunities for observing the pulmonary irritation, and its consequences, produced by an atmosphere loaded with particles of cotton, &c.

"The dust and filaments which are necessarily inhaled in these occupations, occasion considerable pulmonary irritation. In this example, as in others, however, is displayed that peculiar law of structure, by which it insensibly undergoes changes which enable it to endure the presence of a foreign or noxious substance, without suffering the ordinary functional derangement; and a great proportion of the operatives engaged in cotton spinning suffer little, if at all, from the foreign particles which they inspire during twelve hours in the day. The diseases which arise from the circumstances we have described, are chronic and subacute bronchitis—a state of the pulmonary structure in which the signs of inflammatory action are less evident, but in which the chief symptoms indicate a great irritability of the lungs; and these complaints *occasionally* terminate in phthisis.

A chronic inflammation of the mucous membrane of the bronchi, is a common disease amongst those employed in the most dusty rooms of cotton mills. The patients cough and expectorate frequently through the day, whilst engaged in their employment, and especially on awakening in the morning; their respiration is oppressed by inconsiderable exertion; the voice becomes hoarse and harsh; and their strength and health are gradually impaired. Appetite fails, and the digestive function becomes disordered, and, on slight exposure to cold, their symptoms are aggravated, or acute bronchitis occurs, which obliges them to seek medical aid. These cases are often prolonged, and pass through stages of suffering and emaciation which reduce the patient's health to the lowest ebb."

The cough becoming gradually more and more harrassing, the patient applies for medical assistance. There is sometimes expectoration, but the cough is often dry and short, with a diffused and obscure sensation of uneasiness beneath the sternum. On sudden exertion a pectoral oppression ensues, apparently arising from inability to dilate the chest fully in ordinary inspirations. There is little febrile action. On the application of the stethoscope, there is no râle to be heard—and the respiratory murmur is scarcely puerile. Acute bronchitis is easily kindled up by the ordinary causes.

"In families in which consumption has appeared to be hereditary, inasmuch as several of their members have, in succession, become the victims of this insidious disease, I have observed that it has, in the higher ranks also, sometimes been preceded by a state of the pulmonary structure resembling that which I have described, but whose origin could be attributed to no apparent external cause. Conceiving it important to recognize this state early, in order that its progress may be arrested ere fatal mischief has occurred, I shall attempt to delineate the general features of some cases which I have minutely watched, and to which the practice of most will soon furnish them with a parallel.

In families which exhibit distinct evidences of scrophula, one or more of the members not rarely become the victims of tubercular consumption. Shortly after such a catastrophe, the apprehensions of the survivors are awakened to a jealous anxiety concerning the slightest symptoms of indisposition, as they frequently experience a gloomy presentiment that they shall perish by the same fate. A slight cough excites more than ordinary alarm, and the physician is summoned to witness symptoms which might otherwise have been overlooked and neglected. In such constitutions, Phthisis frequently commences most insidiously. The patients are disturbed by a peculiar, short, irritable cough, which is excited by speaking and by slight exertion, but is at first accompanied with little or no disturbance of the respiratory function, and with no expectoration. The health slowly declines; the patient becomes weaker, and gradually more indisposed for exercise, and the pulse is occasionally rapid. After some time, febrile paroxysms may ensue, but are seldom sudden in their accession, or well marked in their progress; though, at their height, the pulse is frequent and sharp. The disease may be fatal, and little or no expectoration occur. In this state, the practitioner may be exceedingly perplexed in forming his diagnosis. The stethoscopic signs often remove all doubt concerning the nature of the disease. The existence of a general harsh, puerile respiration is the most common sign; but sometimes bronchophony, accompanied by bronchial respiration, and a dull sound on percussion, is discovered beneath the clavicles; or a very slight and distant intercurrent crepitous râle may be distinguished, in exceedingly limited portions of the pulmonary structure. This last sign, combined with a distinct and general puerile respiration, may be accepted as very strong evidence in favour of the existence of miliary tubercles.

A year may elapse, the symptoms continuing unchanged, or greater constitutional irritation may have supervened; with more frequent febrile paroxysms, and a more constant rapidity of the pulse—but these signs are often absent. The patient may in this way gradually become emaciated, and, in the last stages, only, may hectic, copious expectoration, or colliquative diarrhœa occur. At other times, acute bronchitis is suddenly developed, without any evident external cause. The pulse is rapid, full and harsh—the skin hot—the breathing much disturbed—the expectoration copious, and resembling that of ordinary inflammation of the bronchi. The puerile respiration may be obscured by the sonorous mucous rales which pervade the lungs. The chief signs which enable the intelligent practitioner to distinguish this disease from ordinary inflammation of the bronchi, are, that *it baffles all the ordinary remedies*. Though antiphlogistic means are freely employed, the pulse maintains its frequency, sharpness, and irritability; and blisters and medicinal agents seem to have no influence over the progress of the complaint. It often terminates fatally in three weeks or a month after the symptoms indicating acute disease appear, or the tubercles soften, and caverns are discovered beneath the clavicles in an exceedingly short space of time.

In these cases, distinct evidence may sometimes be obtained of the existence of extensive tubercular deposition, before the accession of acute symptoms. The patient may even expire without any symptoms excepting a dry short cough, and general constitutional irritation; and when the tubercles excite bronchitis, it evidently arises from some internal cause, and occasions such rapid maturation and softening of the tubercular deposition as to prove speedily fatal. The previous cough must be accepted as evidence of serious pulmonary changes, and of a consequent irritability of the lungs, which deserves watchful scrutiny and careful treatment.

The chronic bronchitis which the spinners suffer, occasionally, though not frequently, terminates in phthisis. I have however, seen well marked examples of tubercular consumption thus induced, but have been unable to detect any peculiarity in its features, by which it may be distinguished from some of those Protean forms of this malady, which occur when it arises from other causes. My able colleague Dr. Phillips has been inclined to think that sudden inflammation and the consequent abrupt termination of the disease, whilst the miliary tubercles are scattered in an unaggregated and unsoftened state through the structure of the lungs, is more frequent in spinners' than in ordinary phthisis. My own observations, on the contrary, have afforded no example in support of this opinion. The number of the cases which I have witnessed, though sufficient to establish the source in which the disease originated, has not, however, been so extended as to enable me to draw conclusions concerning its progress, from a wide comparison of individual examples."

Our author observes that, although scrofula is a powerful predisposing cause, it is by no means essential to the production of phthisis. He thinks that local irritation long kept up will induce the disease in the soundest constitution. That peculiar cachexia which is induced by protracted labour, in unhealthy occupations in large cities—imperfect nutrition—dissolute habits—and mental anxiety, is apt to lead to bronchitis—and ultimately to tubercular consumption.

"The spinners' phthisis is, I conceive, seldom or never produced by the impaction of foreign molecules in the pulmonary tissue, but is a consequence of the morbid actions induced by their constant contact with the mucous membrane. The particles and filaments, with which the air is clouded, are inhaled; they irritate the bronchi, and occasion chronic inflammation, or they excite, in certain constitutions, other and more obscure trains of unhealthy vascular motions, which sometimes terminate in the secretion of miliary tubercles. These affections seldom or never in their origin assume an acute form; they occur chiefly in constitutions weakened by general depressing causes, but an hereditary scrophulous taint does not seem to be necessary to their production. Irritation, when intense and long continued, will triumph over the energies of the most florid health; but all those agents which depress the vigour of the vital powers tend to modify those vascular actions which are excited by local irritation, in such a manner, as to occasion the secretion of tubercles."

Dr. Kay promises to recur to this important investigation at some future time, and we hope he will keep his promise.

---

